# Supplementary figures and images for: One dose of COVID-19 nanoparticle vaccine REVC-128 protects against SARS-CoV-2 challenge at two weeks post-immunization
Source: Emerg Microbes Infect. 2021 Oct 31;10(1):2016–29. doi: 10.1080/22221751.2021.1994354 (PMC8567933; doi:10.1080/22221751.2021.1994354)

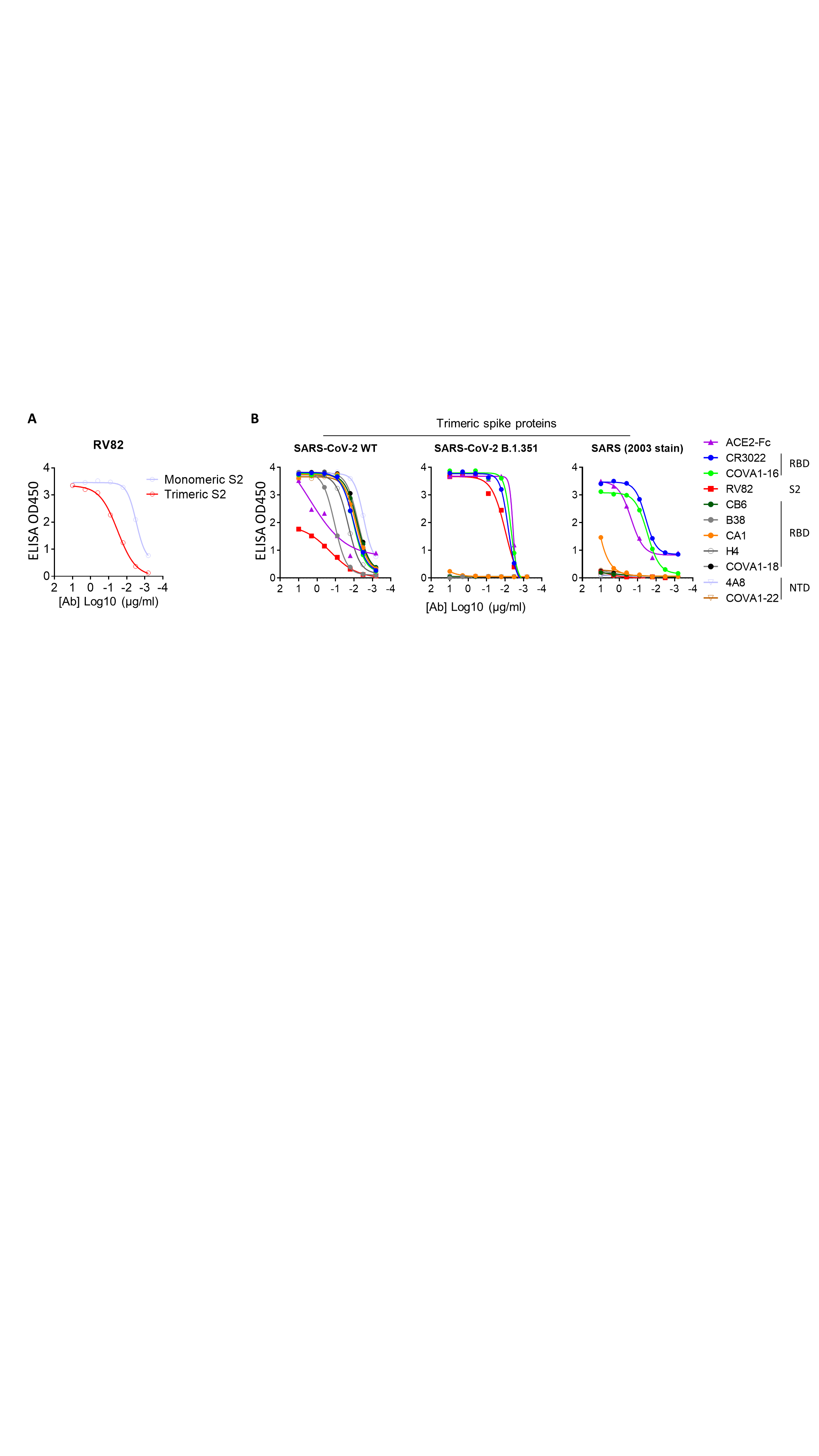

Supplement: Figure_S2.TIF [file TEMI_A_1994354_SM1434.tif]

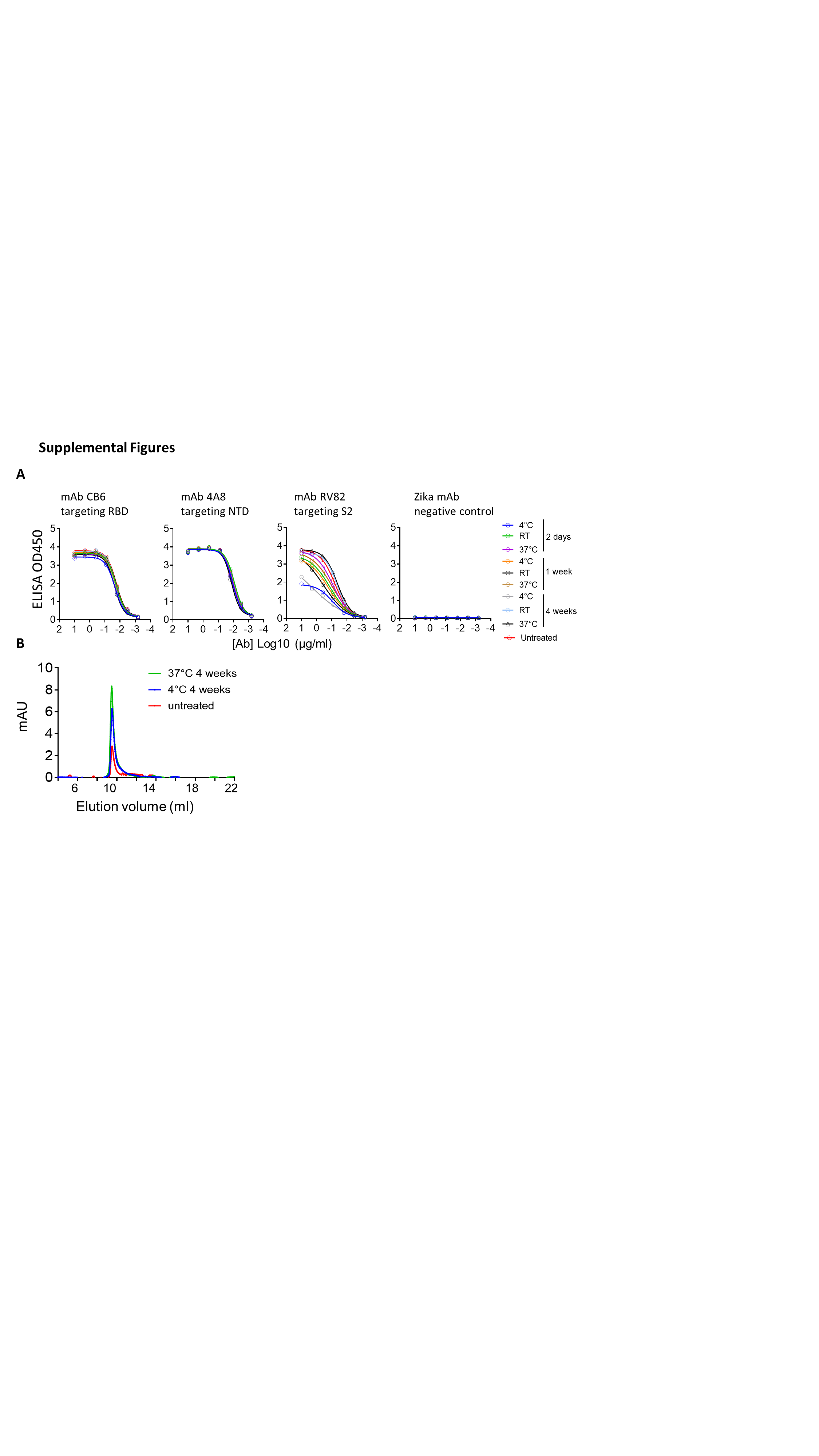

Supplement: Figure_S1.TIF [file TEMI_A_1994354_SM1433.tif]
